# ECMO combined with IABP for the treatment of fulminant myocarditis caused by the targeted drug entrectinib for lung adenocarcinoma: a case report

**DOI:** 10.3389/fcvm.2025.1626318

**Published:** 2025-08-01

**Authors:** Qi Yujuan, Ma Xiaozhong, Wu Zhenhua, Bai Yunpeng

**Affiliations:** ^1^ Department of Intensive Care Unit, Chest Hospital Affiliated to Tianjin University, Tianjin, China; ^2^ Department of Intensive Care Unit, Tianjin Chest Hospital, Tianjin, China; ^3^ Department of Cardiovascular Surgery, Chest Hospital Affiliated to Tianjin University, Tianjin, China; ^4^ Department of Cardiovascular Surgery, Tianjin Chest Hospital, Tianjin, China

**Keywords:** mechanical assistance, entrectinib, cardiogenic shock, fulminant myocarditis, case report

## Abstract

**Background:**

Entrectinib, a recently approved multikinase inhibitor indicated for advanced ROS1-positive non-small cell lung cancer (NSCLC), has demonstrated significant survival benefits in metastatic disease. However, it carries risks of severe cardiotoxicity. We report the successful management of entrectinib-induced fulminant myocarditis using integrated venoarterial extracorporeal membrane oxygenation (V-A ECMO) and intra-aortic balloon pump (IABP) circulatory support.

**Case summary:**

A 71-year-old male diagnosed with lung adenocarcinoma three years ago (ROS1-positive on genetic testing) initiated crizotinib therapy. One week prior to admission, surveillance chest computed tomography(CT) revealed disease progression with increased tumor burden, prompting transition to entrectinib. Seven days post-treatment initiation, he presented to our emergency department with acute-onset palpitations, dyspnea, dizziness, and nausea. Emergent coronary angiography excluded significant stenosis. The patient subsequently developed frequent ventricular premature complexes(VPCs), cardiogenic shock (serum lactate 2.6 mmol/L), and acute heart failure. Absent cardiac history and negative viral serology supported a diagnosis of drug-induced fulminant myocarditis. V-A ECMO with IABP support was emergently instituted. Remarkable recovery ensued: cardiac function normalized by day 3 (ECMO decannulation), followed by extubation and IABP removal on day 5.After 12 days of hospitalization, the patient was discharged. Ejection fraction (EF) recovered from 10% at admission to 61% at discharge. Follow-up demonstrates sustained cardiac function comparable to discharge status.

**Conclusion:**

Entrectinib demonstrates potential cardiotoxicity in lung adenocarcinoma therapy, necessitating prospective studies to quantify this risk. During treatment, multidisciplinary team (MDT) collaboration is essential for rigorous cardiac function surveillance. This case establishes V-A ECMO with IABP as an effective salvage therapy for drug-induced fulminant myocarditis.

## Introduction

Entrectinib is a potent oral tyrosine kinase inhibitor targeting tropomyosin receptor kinases (TRK A/B/C), ROS1, and anaplastic lymphoma kinase (ALK). It has demonstrated significant clinical activity and favorable tolerability in solid tumors harboring these gene fusions (1). It has been approved for patients with ROS1-positive NSCLC and can significantly prolong the survival period of patients (2–4). Cardiotoxicity represents a serious adverse effect associated with this agent, commonly manifesting as QTc interval prolongation and heart failure (5, 6), However, fulminant myocarditis remains exceptionally rare. We present a case of ROS1 fusion-positive lung adenocarcinoma in which entrectinib therapy precipitated drug-induced fulminant myocarditis.

## Case presentation

A 71-year-old male patient presented to the respiratory department three years ago with shortness of breath. CT and PET-CT imaging performed at that time confirmed a clinical TNM stage IVA tumor, with no indication for surgical intervention. Lung tissue pathology confirmed a diagnosis of lung adenocarcinoma. Immunohistochemical staining results were: CK7(+), TTF-1(+), NapsinA(+), P40(-), P63(1+), CK50/6(-), Ki-67 (∼3%+). Genetic testing revealed a ROS1-positive mutation. ROS1-targeted therapy with crizotinib was recommended and initiated at 250 mg twice daily. Having received crizotinib for nearly three years, the patient underwent a follow-up chest CT at an external hospital one week ago that demonstrated tumor enlargement compared to baseline. Consequently, the oncologist discontinued crizotinib and initiated entrectinib—an alternative targeted therapy—at 600 mg orally once daily.

One week later, the patient developed acute-onset palpitations, shortness of breath, dizziness, and nausea without fever, cough, or purulent sputum. Due to persistent symptoms without improvement, he presented to our emergency department. Clinical assessment revealed the following vital signs: blood pressure 92/46 mmHg, heart rate 80 bpm, respiratory rate 27/min, SpO₂91% on room air, and temperature 36.6°C.

**Figure 1:**
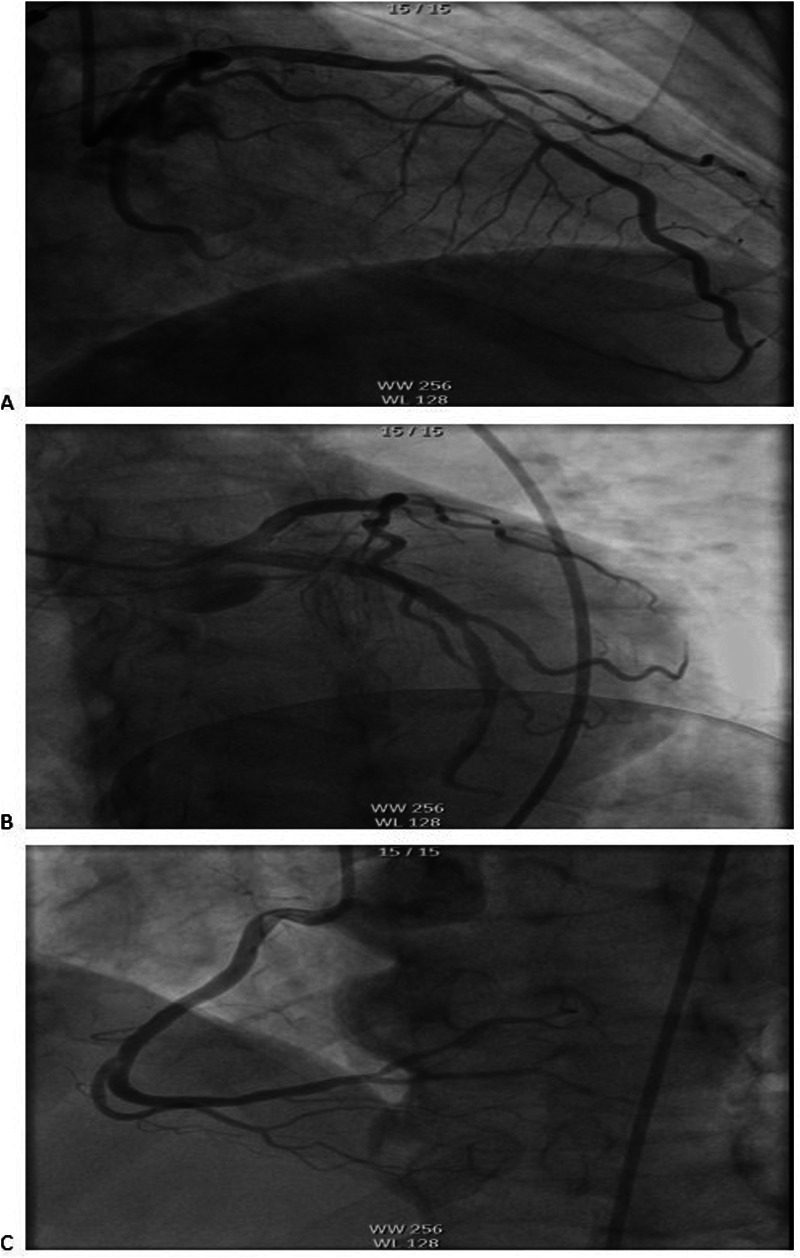
Coronary angiography showed no obvious stenosis in the coronary arteries: **(A)** LAD, **(B)** LCX, **(C)** RCA. LAD, left anterior descending artery; LCX, left circumflex artery; RCA, right coronary artery.

The electrocardiogram (ECG) revealed VPCs, prolonged QTc interval, and ST-T wave changes. Portable transthoracic echocardiography (TTE) revealed global ventricular hypokinesis. Laboratory findings included: Hypersensitive troponin T (hs-TnT): 0.191 ng/ml (ref. ≤0.014 ng/ml); Troponin I (TnI): 0.12 ng/ml; Creatine kinase-MB (CK-MB): 4.3 ng/ml; B-type natriuretic peptide (BNP): 443 pg/ml (ref. <100 pg/ml); D-dimer:526 ng/ml. During emergency department evaluation, the differential diagnoses included coronary artery disease, viral myocarditis, and entrectinib-induced myocarditis. Emergent coronary angiography demonstrated no significant stenosis in the three major coronary vessels: the left anterior descending artery (LAD), left circumflex artery (LCX), and right coronary artery (RCA) (Figure 1).

The patient developed frequent VPCs and cardiogenic shock with echocardiography confirming diffuse biventricular hypokinesis (EF 10%) and elevated serum lactate (2.6 mmol/L), prompting emergent initiation of IABP support, continuous lidocaine infusion at 1–2 mg/min, and vasoactive therapy with adrenaline (0.5–1 μg/kg/min) plus norepinephrine (0.5–1 μg/kg/min); however, hemodynamic instability persisted (BP 70/40 mmHg) with progressive lactic acidosis (peak 3.6 mmol/L), refractory arrhythmias, and worsening cardiac dysfunction, ultimately necessitating emergent endotracheal intubation and V-A ECMO support.

**Figure 2:**
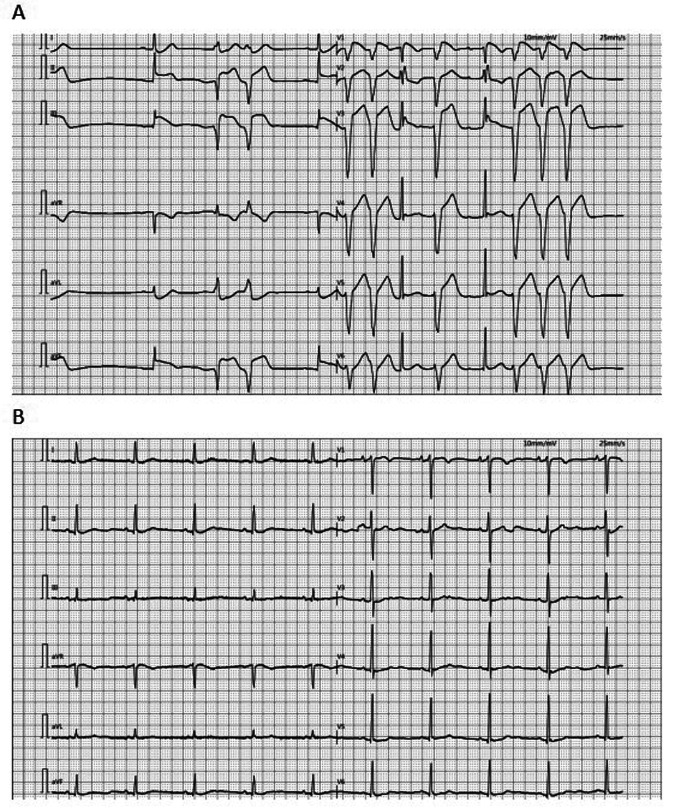
ECG: **(A)** the ECG of the patient at admission showed ventricular premature contractions, prolonged QTc and changes in the ST-T segment. **(B)** The ECG of the patient transferred back to the general ward after the operation showed sinus rhythm. ECG, electrocardiogram.

**Figure 3:**
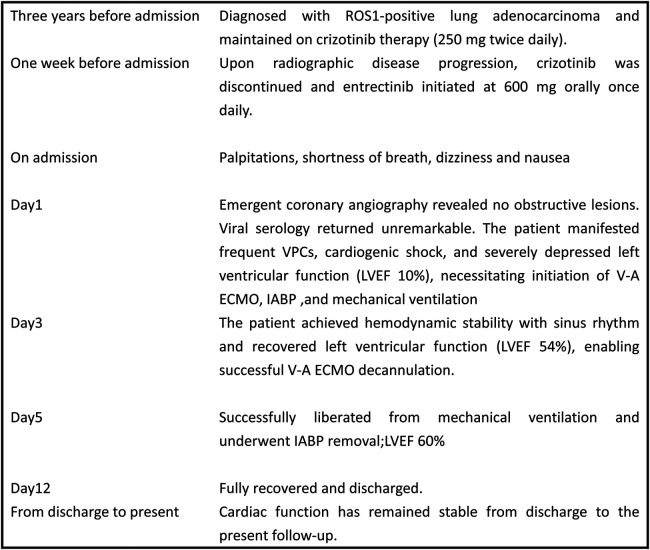
Case timeline. ECMO, extracorporeal membrane oxygenation; IABP, intra-aortic ballon pump; LVEF, left ventricular ejection fraction.

In China, viral infection represents the most common etiology of adult fulminant myocarditis. Therefore, comprehensive virological testing was performed, including:Human cytomegalovirus (CMV) DNA、Epstein–Barr virus (EBV) DNA、Respiratory viral serology panel(Human respiratory syncytial virus IgM 、Coxsackievirus IgM 、Adenovirus IgM and Chlamydia pneumoniae IgM) and SARS-CoV-2 RNA/PCR. All test results returned within normal reference ranges.The patient's family declined endomyocardial biopsy due to concerns regarding bleeding and myocardial injury. Based on comprehensive inpatient investigations, laboratory findings, and medical history—notably the absence of any medications other than entrectinib prior to admission—a diagnosis of drug-induced fulminant myocarditis was established. Post-intervention recovery progressed as follows: left ventricular ejection fraction (LVEF) measured 18% on postoperative day (POD) 2; by POD 3, the patient regained normal consciousness and limb strength with hemodynamic stability maintained without vasopressors or antiarrhythmics, demonstrating sinus rhythm (lactate 1.1 mmol/L, LVEF 54%), prompting discontinuation of V-A ECMO. On POD 5, echocardiography revealed left atrial diameter 38 mm, left ventricular diameter 47 mm, and LVEF 60% with stable circulation and unchanged oxygenation, leading to extubation and IABP removal. Follow-up echocardiography on POD 9 showed LA 37 mm, LV 50 mm, and LVEF 61%, with normal electrocardiographic findings (Figure 2B). The patient was discharged on POD 12. Immunomodulatory therapy comprised intravenous methylprednisolone 40 mg three times daily for 3 days, reduced to 40 mg twice daily for 3 days before discontinuation, supplemented with coenzyme Q10 daily until discharge. Following multidisciplinary team consensus, entrectinib was permanently discontinued given this life-threatening cardiotoxicity. Notably, one-month outpatient echocardiographic follow-up demonstrated complete cardiac recovery with LVEF at 65%. Figure 3 provides a schematic summary of the clinical course and management timeline.

## Discussion

ROS1 gene fusion represents an established oncogenic driver, occurring in 1%-2% of NSCLC cases. Oral small-molecule tyrosine kinase inhibitors (TKIs) targeting ROS1 fusion oncoproteins have revolutionized treatment paradigms for metastatic ROS1-positive NSCLC, significantly improving patient prognosis. Entrectinib—a CNS-active multikinase inhibitor targeting ROS1, TRK A/B/C, and ALK—received U.S. Food and Drug Administration (FDA) approval in 2019 as first-line therapy for ROS1-positive metastatic NSCLC (7). In pivotal clinical trials involving adult patients with treatment-naïve locally advanced or metastatic disease, entrectinib demonstrated significant therapeutic efficacy across ROS1-positive NSCLC and NTRK fusion-positive solid tumors—regardless of baseline central nervous system (CNS) metastasis status (8).

One study has documented cardiotoxicity associated with several NSCLC-targeted therapies, including conduction abnormalities, QTc interval prolongation, ventricular arrhythmias, and heart failure (9). When evaluating the safety and efficacy of entrectinib for ROS1 fusion-positive NSCLC, Drilon et al. reported that 94% of patients experienced ≥1 treatment-related adverse event (TRAE). The most common grade 1–2 TRAEs were dysgeusia, dizziness, and constipation; grade 3 weight increase was observed. Grade 4 TRAEs were uncommon with no treatment-related deaths. Treatment discontinuation due to TRAEs occurred in 5% of patients, with cardiac events being the most frequent cause (2%) (2). To our knowledge, this represents only the second reported case of entrectinib-induced fulminant myocarditis in NSCLC patients, and notably the first documented case demonstrating both the fastest recovery (ECMO decannulation within 72 h) and longest survival to date.The index case involved a 27-year-old male with lung adenocarcinoma who developed new-onset congestive heart failure two weeks after initiating entrectinib. He progressed to refractory ventricular tachycardia and cardiogenic shock, with endomyocardial biopsy confirming active lymphohistiocytic myocarditis. Following emergent mechanical circulatory support (including V-A ECMO), his hemodynamics stabilized. Successful ECMO decannulation occurred on postoperative day 8 with complete symptomatic resolution and functional recovery to baseline. The patient subsequently declined further anticancer therapy. Four months post-discharge, he developed malignant pericardial effusion and succumbed to disease progression (10).

Per ESC guidelines, pretreatment cardiovascular risk assessment is recommended prior to TKI initiation, including physical examination, blood pressure measurement, 12-lead ECG, TTE, fasting lipid profile, and hemoglobin A1c (HbA1c) quantification (11). Literature review identified three case reports of entrectinib-induced cardiotoxicity occurring within the initial treatment phase (days 3, 14, and 19 post-initiation) (5, 6, 12). Notably, our case manifested symptom onset at one week. These findings underscore the necessity for in-hospital monitoring during entrectinib induction, including continuous vital sign surveillance, serial cardiac biomarker panels, 12-lead ECG, and echocardiographic assessments.In this case, the patient presented with elevated troponin levels and electrocardiographic ST-T segment changes. Emergent coronary angiography was performed to exclude acute coronary syndrome, yielding no obstructive lesions. Viral serology panels returned unremarkable, and combined with the patient's history, viral myocarditis was deemed unlikely. When the patient developed frequent VPCs and cardiogenic shock refractory to medical therapy, with no structural abnormalities detected, a diagnosis of fulminant myocarditis was established. While endomyocardial biopsy remains the gold standard for myocarditis diagnosis (13), this procedure was contraindicated during the patient's hemodynamic instability and deferred due to family concerns regarding procedural risks. Following initiation of IABP and V-A ECMO support, intravenous methylprednisolone was administered to suppress inflammatory cascades. Concurrent myocardial protection strategies included parenteral nutrition optimization to enhance myocardial oxygen delivery while reducing metabolic demand. Remarkably, EF recovered from 10% to 54% by postoperative day 3, permitting successful ECMO decannulation. This rapid hemodynamic recovery demonstrates the efficacy of ECMO-IABP circulatory support in fulminant myocarditis. Given the patient's significant clinical improvement and to minimize invasive interventions, surveillance endomyocardial biopsy was appropriately deferred during convalescence.

Current FDA and European Medicines Agency (EMA) guidelines lack specific recommendations on entrectinib discontinuation during myocarditis. Consequently, an emergency multidisciplinary team (MDT) consultation was convened with intensive care, cardiothoracic surgery, and oncology specialists. Given the documented risks of fatal heart failure and fulminant myocarditis associated with entrectinib—particularly concerning in this elderly patient where continued therapy could precipitate lethal cardiotoxicity—unanimous consensus was reached for permanent discontinuation. This case underscores the critical imperative for cardio-oncology collaboration in cancer care and highlights the necessity for prospective studies elucidating entrectinib's cardiovascular toxicity profile, thereby strengthening evidence-based protocols for cardio-protective oncology practice.

## Data Availability

The original contributions presented in the study are included in the article/Supplementary Material, further inquiries can be directed to the corresponding author.

## References

[1] Drilon A Siena S Ou SI Patel M Ahn MJ Lee J Safety and antitumor activity of the multitargeted pan-TRK, ROS1, and ALK inhibitor entrectinib: combined results from two phase I trials (ALKA-372-001 and STARTRK-1). Cancer Discov. (2017) 7(4):400–9. 10.1158/2159-8290.CD-16-1237 28183697 PMC5380583

[2] Drilon A Chiu CH Fan Y Cho BC Lu S Ahn MJ Long-Term efficacy and safety of entrectinib in ROS1 fusion-positive NSCLC. JTO Clin Res Rep. (2022) 3(6):100332. 10.1016/j.jtocrr.2022.100332 35663414 PMC9160474

[3] Yu Y Fan Y Dong X Li J Yu Y Zhao J Entrectinib versus crizotinib in Asian patients with ROS1-positive non-small cell lung cancer: a matching-adjusted indirect comparison. Lung Cancer. (2024) 198:108018. 10.1016/j.lungcan.2024.108018 39549678

[4] Jiang Q Li M Li H Chen L. Entrectinib, a new multi-target inhibitor for cancer therapy. Biomed Pharmacother. (2022) 150:112974. 10.1016/j.biopha.2022.112974 35447552

[5] Otsu Y Kata Y Takayasu H Inoue S Kaneko T. Entrectinib-induced heart failure in a patient with metastatic lung adenocarcinoma: a case report. Cureus. (2022) 14(12):e32174. 10.7759/cureus.32174 36605067 PMC9808486

[6] Futamura K Hase T Tanaka A Sakai Y Okachi S Shibata H Lethal ventricular arrhythmia due to entrectinib-induced brugada syndrome: a case report and literature review. Int Cancer Conf J. (2023) 12(4):299–304. 10.1007/s13691-023-00620-y 37577345 PMC10421830

[7] Boulanger MC Schneider JL Lin JJ. Advances and future directions in ROS1 fusion-positive lung cancer. Oncologist. (2024) 29(11):943–56. 10.1093/oncolo/oyae205 39177972 PMC11546726

[8] Frampton JE. Entrectinib: a review in NTRK+ solid tumours and ROS1+ NSCLC. Drugs. (2021) 81(6):697–708. 10.1007/s40265-021-01503-3 33871816 PMC8149347

[9] Waliany S Zhu H Wakelee H Padda SK Das M Ramchandran K Pharmacovigilance analysis of cardiac toxicities associated with targeted therapies for metastatic NSCLC. J Thorac Oncol. (2021) 16(12):2029–39. 10.1016/j.jtho.2021.07.030 34418561

[10] Rundhawa G Ali M Jacob R Obeng-Gyimah E Vranian MN. Case report of entrectinib associated fulminant myocarditis. Eur Heart J Case Rep. (2024) 9(1):ytae650. 10.1093/ehjcr/ytae650 39802058 PMC11718392

[11] Lyon AR López-Fernández T Couch LS Asteggiano R Aznar MC Bergler-Klein J 2022 ESC guidelines on cardio-oncology developed in collaboration with the European hematology association (EHA), the European Society for Therapeutic Radiology and Oncology (ESTRO) and the international cardio-oncology society (IC-OS). Eur Heart J. (2022) 43(41):4229–361. 10.1093/eurheartj/ehac244 36017568

[12] Fonseca M Chen DH Walker JM Ghosh AK. Entrectinib-related myocarditis in a young female patient with metastatic non-small cell lung cancer. BMJ Case Rep. (2021) 14(7):e243946. 10.1136/bcr-2021-243946 34315748 PMC8317099

[13] Ammirati E Cipriani M Lilliu M Sormani P Varrenti M Raineri C Survival and left ventricular function changes in fulminant versus nonfulminant acute myocarditis. Circulation. (2017) 136(6):529–45. 10.1161/CIRCULATIONAHA.117.026386 28576783

